# Tailoring nutritional and process variables for hyperproduction of catalase from a novel isolated bacterium *Geobacillus* sp. BSS-7

**DOI:** 10.1186/s12934-016-0410-1

**Published:** 2016-01-14

**Authors:** Baljinder Singh Kauldhar, Balwinder Singh Sooch

**Affiliations:** Enzyme Biotechnology Laboratory, Department of Biotechnology, Punjabi University, Patiala, 147002 Punjab India

**Keywords:** *Geobacillus*, Catalase, Extremophilic, Hydrogen peroxide, Fermentation

## Abstract

**Background:**

Catalase (EC 1.11.1.6) is one of the important industrial enzyme employed in diagnostic and analytical methods in the form of biomarkers and biosensors in addition to their enormous applications in textile, paper, food and pharmaceutical sectors. The present study demonstrates the utility of a newly isolated and adapted strain of genus *Geobacillus* possessing unique combination of several industrially important extremophilic properties for the hyper production of catalase. The bacterium can grow over a wide range of pH (3–12) and temperature (10–90 °C) with extraordinary capability to produce catalase.

**Results:**

A novel extremophilic strain belonging to genus *Geobacillus* was exploited for the production of catalase by tailoring its nutritional requirements and process variables. One variable at a time traditional approach followed by computational designing was applied to customize the fermentation process. A simple fermentation media containing only three components namely sucrose (0.55 %, w/v), yeast extract (1.0 %, w/v) and BaCl_2_ (0.08 %, w/v) was designed for the hyperproduction of catalase. A controlled and optimum air supply caused a tremendous increase in the enzyme production on moving the bioprocess from the flask to bioreactor level. The present paper reports high quantum of catalase production (105,000 IU/mg of cells) in a short fermentation time of 12 h. To the best of our knowledge, there is no report in the literature that matches the performance of the developed protocol for the catalase production. This is the first serious study covering intracellular catalase production from thermophilic genus *Geobacillus*.

**Conclusions:**

An increase in intracellular catalase production by 214.72 % was achieved in the optimized medium when transferred from the shake flask to the fermenter level. The extraordinary high production of catalase from *Geobacillus* sp. BSS-7 makes the isolated strain a prospective candidate for bulk catalase production on an industrial scale.

## Background

Industrial demand for microbial enzymes has emerged as the fastest growing market and catalase is one of them. The industrial enzyme market across the globe is growing at a pace with a total turnover of about $3.3 billion in 2010 which is expected to touch $7.1 billion in 2018 [1]. Catalase (EC 1.11.1.6) is one of the important enzyme used in the degradation of hydrogen peroxide into dioxygen and water [2]. This ubiquitous enzyme, found in three forms, has been used for a number of industrial, diagnostic and medical applications. It is produced and purified from a number of microorganisms, plants and animals [3, 4]. Hydrogen peroxide (H_2_O_2_), a strong oxidant, is widely used as a washing reagent in semiconductor factories and as a bleaching agent in textile industries. This industrial wastewater containing H_2_O_2_ needs to be treated to remove H_2_O_2_ as it adversely affects the surrounding flora and fauna [5]. The use of treatment processes employing chemicals, such as sodium hydrogen sulphite, have several disadvantages as the chemicals itself are highly toxic and damages activated sludge which further adds to waste disposal problems. The approach involving enzymatic degradation of H_2_O_2_ with catalase is devoid of the aforementioned drawbacks, and therefore, is a safer alternate to the chemical treatment approach.

**Fig. 1:**
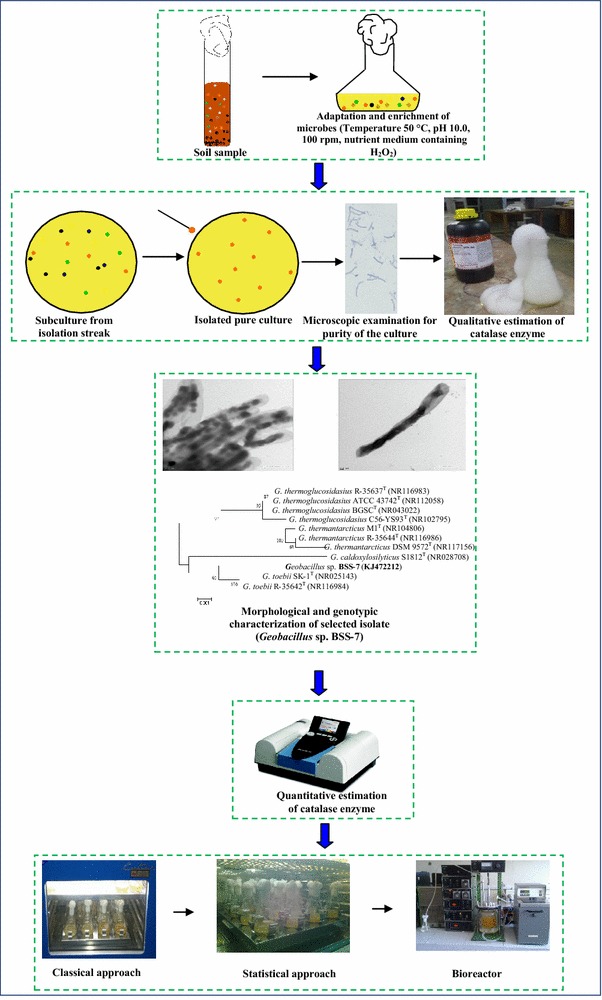
Soil to industrial applications: Schematic overview of microbial bioprocess for catalase production

Many strategies have been employed for the synthesis of catalase from microorganisms, which include optimization of critical factors, induction by H_2_O_2_, mutagenesis and other advanced genetic engineering approaches. However, all of these strategies failed to produce commercially viable catalase from microorganisms under adverse industrial process conditions [6, 7]. The harsh industrial conditions to which enzymes are subjected provide impetus for isolation of extremozyme producing extremophiles by employing specific strategies. Hence, the present study was carried out to explore catalase producing potential of an endospore forming newly isolated and adapted extremophilic bacterium *Geobacillus* sp. BSS-7 under harsh industrial conditions. The strain is deposited at the Institute of Microbial Technology, Chandigarh under an accession no. MTCC 5873. The 16S rRNA sequence of this isolate has been submitted to the Genbank (NCBI) under an accession number KJ472212. In addition, this piece of research focuses on enhancing the microbial production of catalase by tailoring nutritional and multiple bioprocess parameters at the flask level (Fig. 1). The design of the study was further refined to improve the enzyme productivity using statistical tools. The scale up studies for catalase production was also carried out at bioreactor level. In the present investigations, a significant catalase yield of 105,000 IU/mg was achieved in a very short fermentation time of 12 h using above mentioned strategies. A comparative analysis of scientific literature reveals that the catalase yield in the present developed process is much better than other reported microbial sources like *Rhizobium radiobacter* (30,420 IU/mg in 20 h) [8], *Exiguobacterium* *oxidotolerans* T-2-2^T^ (16,000 IU/mg in 24 h) [9], *Thermoascus aurantiacus* (5100 IU/mg in 20 days) [10] and *Vibrio rumoiensis* (4092 IU/mg in 48 h) [11].

## Results and discussion

### Catalase production with respect to growth pattern of *Geobacillus* sp. BSS-7

**Fig. 2:**
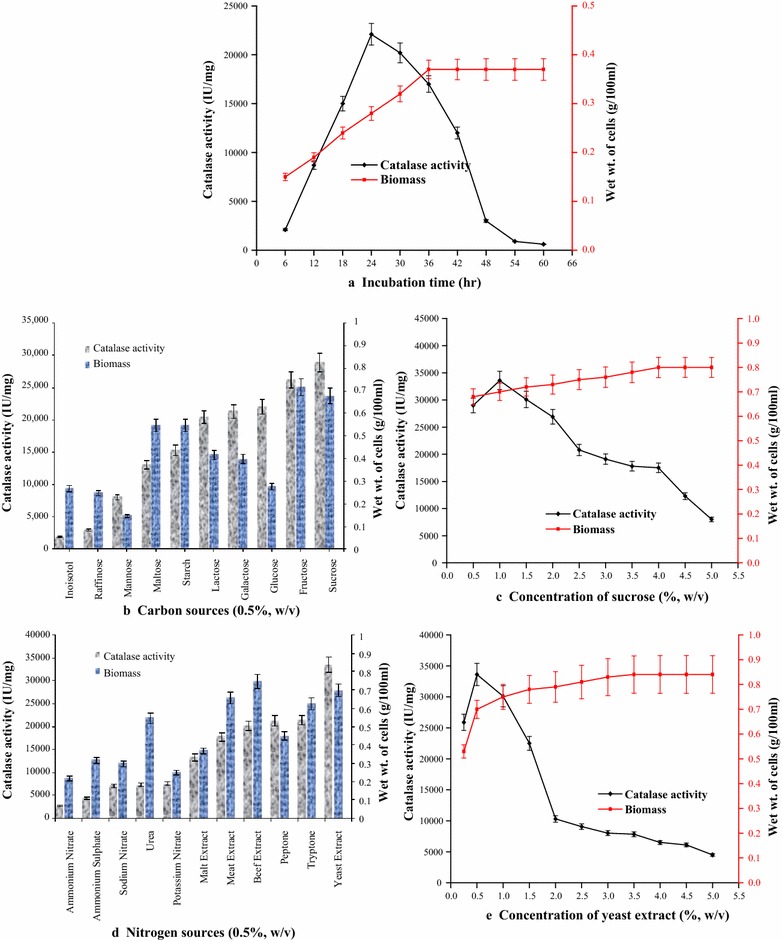
**a** Growth *curve* of *Geobacillus* sp. BSS-7; influence of medium constituents on catalase production **b** carbon sources, **c** sucrose concentration, **d** nitrogen sources, **e** yeast extract concentration

Catalase activity was recorded only in sonicated biomass of *Geobacillus* sp. BSS-7 cells separated from the fermented broth and no extracellular catalase activity was found in the supernatant. This proves that *Geobacillus* sp. BSS-7 produces intracellular catalase only. Production of biocatalysts is growth associated in nature and enzyme biosynthesis begins on attaining the log phase of growth [12]. It was analysed from the growth curve that *Geobacillus* sp. BSS-7 reached its stationary phase after 36 h and thereafter, a decline in the growth and catalase activity was recorded. However, the maximum catalase activity of 22,100 IU/mg was obtained before the onset of stationary phase (Fig. 2a). Similar observations were also reported by other research groups where maximum catalase production has been achieved from *Bacillus halodurans* LBK 261 [13] and *Bacillus* *maroccanus* [14] after 16 and 24 h of incubation, respectively.

The effect of H_2_O_2_ (0.05–1.0 mM) as an inducer of catalase enzyme was also investigated. It was found that there is no inducing effect of H_2_O_2_ on the enzyme synthesis. This clearly establishes the constitutive nature of the enzyme in *Geobacillus* sp. BSS-7. Takebe and co-workers [15] have also reported constitutive nature of the catalase enzyme in *Exiguobacterium oxidotolerans* cells.

### Tailoring of nutrient requirements for catalase production at shake flask level

#### Influence of carbon source

Tailoring of microbial fermentations has been extensively used to increase the yield and productivities of large number of bioprocesses [16]. All the tested carbon sources were found to support bacterial growth and enzyme synthesis, however sucrose was adjudged as the best source for the catalase synthesis (29,000 IU/mg of cells) from *Geobacillus* sp. BSS-7. The order of catalase production from tested carbon sources was sucrose > fructose > glucose > galactose > lactose > starch > maltose > mannose > raffinose > inoisotol (Fig. 2b). Raffinose, mannose and inoisotol were found to be ineffective for the catalase production in comparison to other studied carbon sources. It is pertinent to mention that enhanced catalase production from *Alternaria* *alternata* [17], *Septoria tritici* [18] and *Rhodotorula glutinis* [19] has also been obtained by using sucrose in the production medium by other workers.

The concentration of sucrose in the production medium has a significant effect on the catalase activity. The increase in sucrose concentration in medium up to 1 % (w/v) causes an increase in the catalase production (33,600 IU/mg of cells) and thereafter, the enzyme activity declined (Fig. 2c).

#### Influence of nitrogen source

A comparable catalase production was observed when yeast extract, tryptone, peptone and beef extract were supplemented in the nutritional medium, however the maximum catalase production (33,600 IU/mg of cells) was observed with the yeast extract (Fig. 2d). It was also observed that complex nitrogen sources proved better than the inorganic ones for the production of enzyme. This may be due to the release of acidic ions from inorganic nitrogen sources after their utilization by microorganisms. The high acidic conditions may inhibit the growth and metabolic processes of the microbes and hence the synthesis of enzyme [20]. Yeast extract has also been adjudged as a good nitrogen supplement for the synthesis of catalase from a number of other microorganisms such as *Serratia marcescens* SYBC-01 [21], *Exiguobacterium oxidotolerans* [15] and *Septoria tritici* [18].

Further, the effect of yeast extract concentration on the catalase activity was also studied. A progressive increase in catalase activity (33,600 IU/mg) was recorded with increase of yeast extract concentration up to 0.5 % (w/v) and thereafter, it declined (Fig. 2e). The decrease in enzyme activity above 0.5 % (w/v) yeast extract concentration may be due to its complex nature as some of the ingredients of yeast extract may have caused toxic or inhibitory effect on enzyme synthesis at higher concentrations.

The higher concentration of carbon and nitrogen sources beyond optimum level may also have caused increase in the viscosity of fermentation medium due to the tremendous growth of bacterial biomass, resulting in depletion of oxygen and nutritional imbalance in the medium. This nutritional imbalance and oxygen depletion could have possibly disturbed the cellular machinery and translation processes resulting in lower catalase production.

#### Influence of trace metal salts

The supplementation of the production medium with a variety of trace metal salts (Table 1) did not exert any effect on the production of catalase except BaCl_2_. The results clearly revealed that only BaCl_2_ at a concentration of 0.05 % (w/v) enhances the production of enzyme (36,400 IU/mg of cells). The increase in BaCl_2_ concentration above 0.05 % (w/v) did not exert any significant influence on the enzyme synthesis (Table 1). In contrary, other trace metal salts such as CaCl_2_, ZnSO_4_, CuSO_4_, MnSO_4_, MgSO_4_, FeSO_4_, NiSO_4_, CoCl_2_, HgSO_4_, AgNO_3_, KI, Na_2_SO_3_, Na_2_MoO_4_ inhibit the enzyme production substantially. The addition of salts like NiSO_4_, AgNO_3_, CuSO_4_, CoCl_2_, HgSO_4_ in trace amount led to the complete inhibition of enzyme synthesis and growth of bacterium. This may be due to the change in conformation of enzyme/protein to a less stable form caused by these salts by altering the enzyme surface charge.

**Table 1:** Influence of metal salts on the production of catalase from *Geobacillus* sp. BSS-7

Metal salts (%, w/v)	Catalase activity (IU/mg of cells)	Biomass (g/100 ml)
Control	30,100	0.70
CaCl_2_		
0.001	13,400	0.58
0.01	21,400	0.67
0.05	23,400	0.74
0.10	20,600	0.63
ZnSO_4_		
0.001	11,200	0.49
0.01	24,500	0.70
0.05	12,700	0.57
0.10	8700	0.37
MnSO_4_		
0.001	6500	0.29
0.01	10,200	0.43
0.05	15,900	0.59
0.10	11,200	0.47
MgSO_4_		
0.001	17,800	0.24
0.01	19,300	0.31
0.05	22,300	0.39
0.10	12,300	0.19
FeSO_4_		
0.001	9100	0.41
0.01	17,400	0.47
0.05	12,800	0.37
0.10	10,100	0.30
BaCl_2_		
0.001	23,000	0.64
0.01	31,200	0.72
0.05	36,400	0.85
0.10	16,700	0.36
KI		
0.001	4300	0.30
0.01	10,400	0.41
0.05	11,220	0.49
0.10	1340	0.13
Na_2_SO_3_		
0.001	900	0.09
0.01	5400	0.23
0.05	8300	0.34
0.10	4800	0.17
Na_2_MoO_4_		
0.001	13,400	0.50
0.01	21,300	0.63
0.05	20,200	0.49
0.10	8100	0.29

### Optimization of process parameters at shake flask level

#### Influence of pH and temperature

**Fig. 3:**
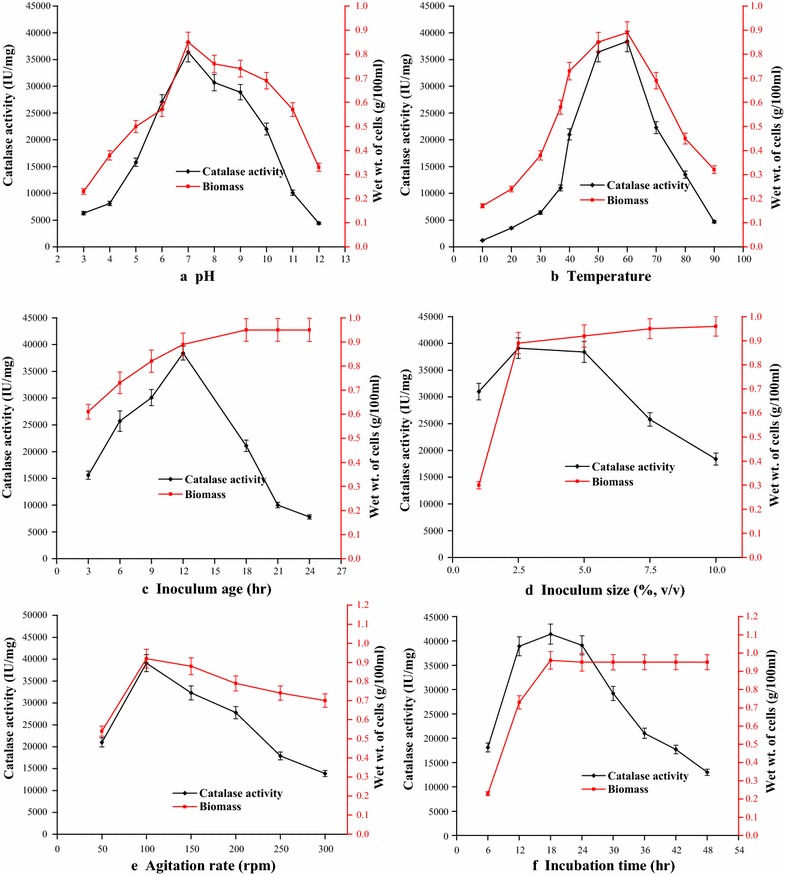
Influence of process parameters on catalase production from *Geobacillus* sp. BSS-7 **a** pH, **b** temperature, **c** inoculum age, **d** inoculum size, **e** agitation rate and **f** incubation time

The pH and temperature of the production medium are critical process variables in enzymatic fermentation processes and their influence on catalase synthesis by *Geobacillus* sp. BSS-7 was investigated. The results revealed an enhancement in catalase and biomass production with an increase in pH of the fermentation medium up to pH 7.0 (36,400 IU/mg of cells), and thereafter, it declined (Fig. 3a). The low enzyme production at lower and higher pH values may be due to alteration in the ionization of nutrient molecules, which thereby reduce their availability to the organism and hence disturb the ionic balance. In addition, drastic variations in pH can also harm the microbial cells by disrupting the plasma membrane and disturbing their metabolism. It is clear from the Fig. 3a that *Geobacillus* sp. BSS-7 was able to grow and produce catalase at a broad pH range. There are scarce reports on the production of catalase at higher pH (>8.0) values and these are from cultures of other genera i.e. *Bacillus* sp. SF [23] and *Oceanobacillus oncorhynchi* [26].

An increase in biomass along with catalase activity was observed with increase in temperature up to 60 °C (38,400 IU/mg of cells) and thereafter, a progressive decline in both biomass and catalase synthesis was recorded (Fig. 3b). However, the said strain was able to grow and produce catalase over a wide range of temperature from 10 to 90 °C. To best of our knowledge, this is the first report from genus *Geobacillus*, where bacterium is able to grow above 80 °C and below 30 °C.

The rate of enzymatic reaction in the cells increases with the increase in temperature till an optimum temperature is reached. Beyond the optimum temperature, the enzyme inactivation occurs due to protein denaturation, which ultimately affects the cell growth and productivity. The lower catalase production at high temperatures could also be due to the decreased oxygen solubility in the medium. Further, the gelling of plasma membrane at low temperature results in slowdown of transport processes and metabolism in the microbial cells. The majority of catalase producing microorganisms reported in the literature produce catalase below 50 °C, and that too at a neutral pH. There is only one study demonstrating the production of an enzyme at 65 °C by a bacterial strain *Bacillus* sp. SF [23].

**Fig. 4:**
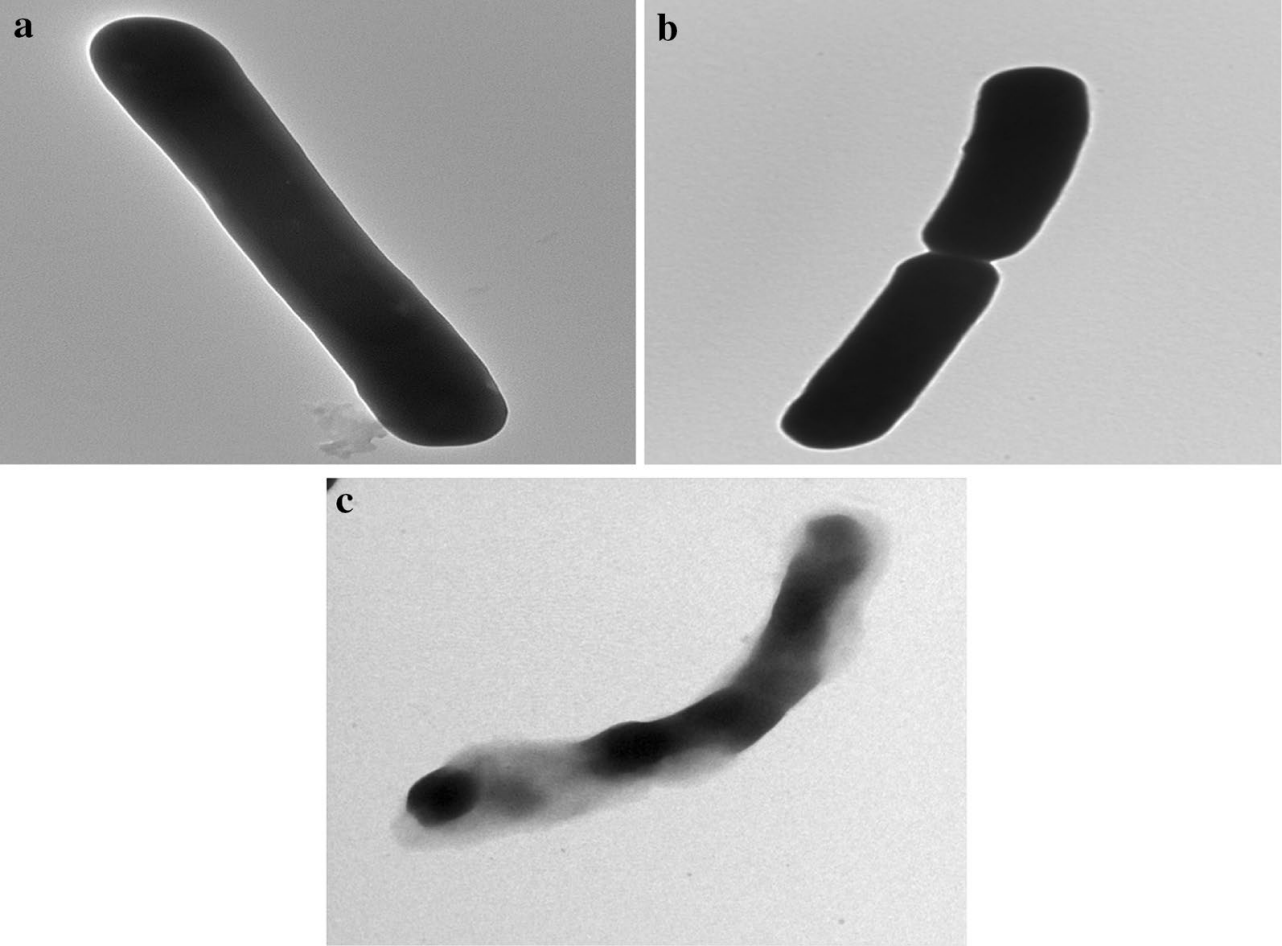
Transmission Electron Micrographs of *Geobacillus* sp. BSS-7 showing cell morphology at **a** 20 °C, **b** 50 °C, **c** 80 °C

The cellular morphology of *Geobacillus* sp. BSS-7 at different temperatures (20, 50 and 80 °C) was also studied by Transmission Electron Microscopy (FEI, Tecnai, G2 F20, USA) and shown in Fig. 4a–c. Most of the cells were observed under active cell division when grown at 50 °C (Fig. 4b), whereas, bacterial cells with thick cell membrane were seen at 20 °C and only few cells were seen under division phase at this temperature (Fig. 4a). A noteworthy change in cellular morphology of *Geobacillus* sp. BSS-7 was observed when the cells were grown at 80 °C (Fig. 4c). The bacterial cells were seen as intracellular oval endospore forming rod shaped structures at this temperature. These endospores would have possibly the mechanism for *Geobacillus* sp. BSS-7 to survive under stress conditions. Limited literature is available on the cellular morphology of *Geobacilli* under extreme conditions but the presence of long endospore forming rod shaped cells of *G*. *thermoleovorans* at high temperatures was also observed by Marchant and co-workers [24, 25].

#### Influence of inoculum age and size

Inoculum age and size were observed to be significant parameters that exert a strong effect on the catalase production. The catalase production reached up to a maximum level of 38,400 IU/mg of cells with a 12 h old inoculum (Fig. 3c). In contrast, a lower catalase activity was obtained by using cells of inoculum age below 12 h, which could be due to the fact that bacterial cells might not have reached the exponential growth phase at that time. Keeping this in view, 12 h old culture was selected for further studies. An increase in catalase production (39,100 IU/mg of cells) was also recorded with the increase in inoculum size up to 2.5 % (v/v), and thereafter, it decreased (Fig. 3d). Hence, the inoculum size of 12 h old culture containing 1.2 × 10^8^ cells/mL was adjusted to achieve the maximum yield of enzyme.

A tremendous growth of bacteria with higher inoculum size results in nutritional imbalance due to stiff competition for nutrients between the cells. This ultimately leads to a decrease in the enzyme production capacity. On the other hand, low enzyme production below 2.5 % (v/v) inoculum is probably due to insufficient biomass for maximum utilization of nutrient molecules and production of catalase. An inoculum size of 2 % (v/v) for the production of catalase from *Pseudomonas* *putida* and *Bacillus* *subtilis* has been reported [22, 27].

#### Influence of agitation rate

The agitation of growth medium exerts a significant influence on the catalase synthesis as it affects the mixing of nutritional components and exchange of gases which further controls the availability of nutrients and oxygen to microbial cells. A progressive increase in enzyme activity (39,100 IU/mg of cells) was recorded with agitation rate up to 100 rpm, and thereafter, a decline in catalase synthesis was observed (Fig. 3e). Decreased enzyme activity at lower agitation rate may be due to inadequate mixing and supply of media components, and slow diffusion of gases. Whereas, decrease in catalase activity at higher agitation rate can be due to the influence of shear stress on bacterial cells. Similar to our observation, 100 rpm agitation rate has been reported suitable for catalase production from *Serratia marcescens* [28] and *Thermus brockianus* [29].

#### Influence of incubation time

The increase in catalase production was achieved with time and the maximum enzyme production (41,400 IU/mg of cells) was observed after 18 h of incubation (Fig. 3f). This trend may be due to an increase in the formation of oxygen radicals as a result of high metabolic rate during exponential growth phase. This enhanced production of reactive oxygen stimulates the catalase synthesis. However, they begin to form spores during late exponential phase (after 18 h) due to decrease in nutrient availability which lead to a decreased metabolism, and hence, a decline in the catalase synthesis was recorded.

### Response surface optimization

Tailoring of bioprocess components by traditional methods involves the change of one variable at a time which is extremely expensive and time consuming. In order to combat this drawback and determine the interaction between different experimental factors, a central composite response design was employed for the optimization of nutrient and process parameters. The observations of CCRD experiments to analyse the effect of six independent variables are shown in Table 2 along with the predicted and observed responses. The experimental results obtained were fitted in a second order polynomial equation. The fitted equation (in terms of coded value) for predicting catalase production after calculating the regression coefficient value was given succeedingly regardless of the significance of the coefficients:

1 $$\begin{aligned} {\text{EA}} & = + 19{,}995.27 + 2312.83\; \times \;{\text{A}} + 3989.95\; \times \;{\text{B}} + 1430. 80\; \times \;{\text{C}} - 732.22\; \times \;{\text{D}} + 3145.41\;\times\;{\text{E}}\\ & + 621.15\; \times \;{\text{F}} - 2849.29\; \times \;{\text{A}}\; \times \;{\text{B}} + 3062.07\; \times \;{\text{A}}\; \times \;{\text{CC}} + 4322.13\; \times \;{\text{A}}\; \times \;{\text{D}} - 418.17\; \times \;{\text{A}}\; \times \;{\text{E}} \\ & - 83.70\; \times \;{\text{A}}\; \times \;{\text{F}} + 5506.09\; \times \;{\text{B}}\; \times \;{\text{C}} + 678.11\; \times \;{\text{B}}\; \times \;{\text{D}} - 1862.18\; \times \;{\text{B}}\; \times \;{\text{E}} + 2647.28\; \times \;{\text{B}}\; \times \;{\text{F}} \\ & - 533.24\; \times \;{\text{C}}\; \times \;{\text{D}} + 3351.46\; \times \;{\text{C}}\; \times \;{\text{E}} - 2339.07\; \times \;{\text{C}}\; \times \;{\text{F}} + 439.34\; \times \;{\text{D}}\; \times \;{\text{E}} - 1545.12\; \times \;{\text{D}}\; \times \;{\text{F}} \\ & + 270.17\; \times \;{\text{E}}\; \times \;{\text{F}} + 1944.21\; \times \;{\text{A}}^{ 2} - 2383.22\; \times \;{\text{B}}^{ 2} + 5516.38\; \times \;{\text{C}}^{ 2} + 8414.95\; \times \;{\text{D}}^{ 2} + 86.68\; \times \;{\text{E}}^{ 2} \\ & + 1576.79\; \times \;{\text{F}}^{ 2} \\ \end{aligned}$$

where A, B, C, D, E and F represents sucrose, yeast extract, barium chloride, inoculum age, inoculum size and time, respectively. The statistical significance of Eq. (1) for response surface quadratic model was tested by ANOVA and results are presented in Table 3. The quadratic model in Eq. (1) with 27 terms have six linear, six quadratic and fifteen two factorial interactions. The insignificant terms were neglected on *p* value basis (*p* values more than 0.0500 were excluded). Then, the model Eq. (1) was amended to reduced fitted model Eq. (2):

2 $$\begin{aligned} {\text{EA}} = & + 19{,}995.27 + 2312.83\; \times \;{\text{A}} + 3989.95\; \times \;{\text{B}} + 1430.80\; \times \;{\text{C}} - 732.22\; \times \;{\text{D}} \\ & + 3145.41\; \times \;{\text{E}} + 621.15\; \times \;{\text{F}} - 2849.29\; \times \;{\text{A}}\; \times \;{\text{B}} + 4322.13\; \times \;{\text{A}}\; \times \;{\text{D}} \\ & {-}{ 83}.70\; \times \;{\text{A}}\; \times \;{\text{F}} + 5506.09\; \times \;{\text{B}}\; \times \;{\text{C}} + 678.11\; \times \;{\text{B}}\; \times \;{\text{D}} - 1862.18\; \times \;{\text{B}}\; \times \;{\text{E}} \\ & + 2647.28\; \times \;{\text{B}}\; \times \;{\text{F}} - 533.24\; \times \;{\text{C}}\; \times \;{\text{D}} - 2339.07\; \times \;{\text{C}}\; \times \;{\text{F}} + 439.34\; \times \;{\text{D}}\; \times \;{\text{E}} \\ & - 1545.12\; \times \;{\text{D}}\; \times \;{\text{F}} + 270.17\; \times \;{\text{E}}\; \times \;{\text{F}} + 1944.21\; \times \;{\text{A}}^{ 2} - 2383.22\; \times \;{\text{B}}^{ 2} + 5516.38\; \times \;{\text{C}}^{ 2} \\ & + 8414.95\; \times \;{\text{D}}^{ 2} + 86.68\; \times \;{\text{E}}^{ 2} + 1576.79\; \times \;{\text{F}}^{ 2} \\ \end{aligned}$$

**Table 2:** Central composite design matrix for experimental and predicted results for catalase activity

Run	Factors^a^						Experimental results	Predicted results
	A	B	C	D	E	F	Catalase activity (IU/mg wet weight)	
1	0.50	1.00	0.01	6.00	5.00	24.00	42,100	42,200
2	1.25	0.63	0.06	12.00	3.00	18.00	19,500	19,400
3	0.50	0.25	0.10	18.00	5.00	12.00	25,600	25,700
4	0.50	0.25	0.01	6.00	5.00	12.00	36,800	35,900
5	1.00	1.00	0.10	12.00	5.00	12.00	46,700	47,400
6	0.50	0.25	0.06	6.00	1.00	24.00	41,900	41,500
7	2.00	1.00	0.01	6.00	1.00	12.00	20,800	20,600
8	1.25	0.63	0.06	12.00	3.00	18.00	19,500	19,400
9	2.00	0.25	0.01	18.00	1.00	24.00	40,400	40,300
10	2.00	0.25	0.10	6.00	1.00	12.00	32,000	32,100
11	2.00	0.25	0.01	6.00	5.00	24.00	35,200	35,400
12	1.25	0.63	0.06	12.00	3.00	18.00	19,500	19,200
13	2.00	0.25	0.01	18.00	5.00	12.00	41,900	41,600
14	0.50	1.00	0.01	18.00	1.00	24.00	38,500	38,200
15	0.50	0.25	0.10	18.00	1.00	24.00	21,800	20,900
16	0.50	1.25	0.01	18.00	5.00	12.00	23,300	23,100
17	0.50	1.00	0.10	6.00	1.00	12.00	42,100	42,000
18	0.50	0.25	0.10	6.00	10.00	24.00	29,300	29,500
19	2.00	1.00	0.10	6.00	1.00	12.00	39,100	39,200
20	0.50	0.25	0.01	18.00	1.00	12.00	30,300	30,000
21	1.25	0.63	0.06	12.00	3.00	18.00	19,500	19,300
22	2.00	1.00	0.10	6.00	1.00	24.00	42,600	42,100
23	1.25	0.63	0.06	12.00	3.00	18.00	19,500	19,400
24	2.00	0.25	0.10	18.00	5.00	24.00	38,000	38,100
25	1.25	0.63	0.06	12.00	3.00	18.00	19,500	19,200
26	2.00	1.00	0.01	18.00	5.00	24.00	32,000	31,700
27	1.25	1.00	0.10	12.00	3.00	18.00	40,200	40,400
28	1.25	1.00	0.10	12.00	3.00	18.00	40,200	40,400
29	1.25	1.00	0.10	12.00	3.00	18.00	40,200	40,400
30	1.25	1.00	0.10	12.00	3.00	18.00	40,200	40,400
31	1.25	0.63	0.06	12.00	3.00	18.00	19,500	19,700
32	1.25	0.63	0.06	12.00	3.00	8.61	24,800	24,600
33	2.42	0.63	0.06	12.00	3.00	18.00	32,500	32,200
34	1.25	0.04	0.06	12.00	3.00	18.00	9400	9000
35	0.25	1.25	0.06	12.00	3.00	18.00	30,000	28,900
36	1.25	1.00	0.10	12.00	3.00	18.00	40,200	40,400
37	1.25	0.63	0.02	12.00	8.00	18.00	28,200	28,000
38	1.25	0.63	0.06	21.39	3.00	18.00	39,500	40,000
39	1.25	0.63	0.06	12.00	3.00	18.00	11,200	11,500
40	1.25	0.63	0.06	12.00	6.13	18.00	42,100	41,900
41	1.25	0.63	0.13	12.00	8.00	18.00	40,500	39,800
42	1.25	0.63	0.06	12.00	3.00	27.39	24,600	24,700

^a^Symbols A, B, C, D, E, F are the same as mentioned in Table 5

**Table 3:** Analysis of variance (ANOVA) and model coefficients estimated by multiple linear regression for response surface quadratic model

Factors^a^	Catalase activity					
	Sum of squares	df	*p* value	Coefficient estimate	Standard error	*F* value
Model	4.857E+009	27	0.0041	19995.27	2563.98	11.76
A	9.897E+007	1	0.0021	2312.83	1968.55	49.7
B	2.955E+008	1	0.0053	3989.95	1968.55	20.08
C	3.788E+007	1	0.0124	1430.80	1968.55	10.04
D	9.9920E+006	1	0.0134	−732.22	1968.55	32.63
E	1.830E+008	1	0.0001	3145.41	1968.55	15.06
F	7.138E+006	1	0.0244	621.15	1968.55	1.34
AB	1.353E+008	1	0.0061	−2849.29	2074.24	19.57
AC	1.562E+008	1	0.2321	3062.07	2074.24	0.81
AD	3.113E+008	1	0.0126	4322.13	2074.24	11.97
AE	2.914E+006	1	0.4421	−418.17	2074.24	0.55
AF	1.167E+005	1	0.0403	−83.70	2074.24	1.98
BC	5.052E+008	1	0.0093	5506.09	2074.24	12.54
BD	7.663E+006	1	0.0042	678.11	2074.24	2.62
BE	5.779E+007	1	0.0002	−1862.18	2074.24	3.21
BF	1.168E+008	1	0.0065	2647.28	2074.24	17.57
CD	4.738E+006	1	0.0036	−533.24	2074.24	20.12
CE	1.872E+008	1	0.2145	3351.46	2074.24	1.37
CF	9.117E+007	1	0.0003	−2339.07	2074.24	17.32
DE	3.217E+006	1	0.0074	439.34	2074.24	5.21
DF	3.978E+007	1	0.0256	−1545.12	2074.24	3.98
EF	1.216E+006	1	0.0001	270.17	2074.24	1.28
A^2^	5.006E+007	1	0.0215	1944.21	2326.81	6.02
B^2^	7.521E+007	1	0.0324	−2383.22	2326.81	8.32
C^2^	4.030E+008	1	0.0339	5516.38	2326.81	8.11
D^2^	9.377E+008	1	0.0031	8414.95	2326.81	41.39
E^2^	99503.63	1	0.0470	86.68	2326.81	2.21
F^2^	3.292E+007	1	0.0669	1576.79	2326.81	1.24
Residual	9.321E+008	13				
Lack of fit	9.321E+008	7				
Pure error	0.00	6				
Core total	6.045E+009	41				

^a^Symbols A, B, C, D, E, F are the same as mentioned in Table 5

It is clearly depicted from Table 3 that the present model is significant. The significance was also recognized from a very low probability value for catalase production (*P*_value_ < 0.05). The *P* values further indicate the pattern of interaction between the coefficients on the basis of the significance of each coefficient. Smaller the *P* value, the more significant is the corresponding coefficient. The coefficient of determination (*R* ^2^) presents a measure of the variability in the actual response values. This variability can be described by the experimental variables and their interactions.

A value of 1 suggests an ideal case at which 100 % of the variation in the experimental value can be explicated by the model. The matching quality of the values acquired by the model was analysed with respect to coefficient of determination (*R* ^2^). The mathematical tuning of those values which generated *R* ^2^ = 0.9876 (98.76 %) for catalase production have revealed that the model is unable to describe only 1.24 % of all the effects, thereby representing it as the best statistical model. The coefficient estimates of Eq. (2) are shown in Table 3. The model *F* values of 11.76 for catalase production imply that the model is significant, where *F* value represents the ratio of mean squares due to regression and mean squares due to error. Adequate precision is a measure of signal to noise ratio (value greater than 4 is desirable) and this ratio of 7.301 indicates an adequate signal for catalase activity. The lack of fit value for catalase activity (9.321E + 008) relative to pure error (Table 3) specifies that the experimental data attained fitted well with the model.

**Fig. 5:**
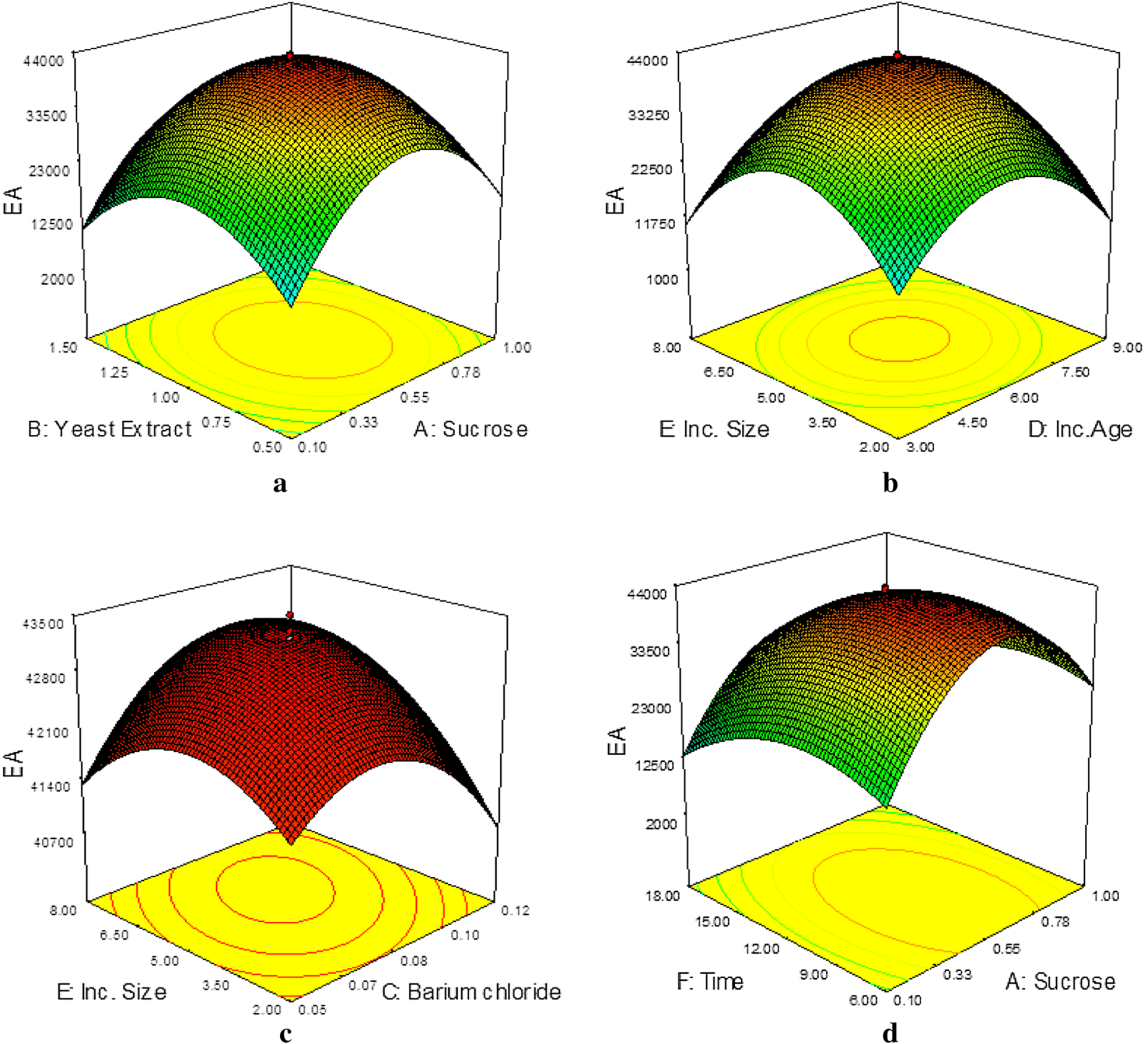
Response surface curves showing the effect of interaction of two factors on catalase production keeping other factors constant **a** sucrose and yeast extract, **b** inoculum size and inoculum age, **c** inoculum size and barium chloride, **d** time and sucrose

The three dimensional response surface graphs (Fig. 5a–d) based on the final model were plotted by keeping four variables at their optimum value while varying the other two variables within their experimental range. The contour curves were used for analysing the effects of different variables on the catalase production. Statistically optimal values of variables were obtained from these curves while moving along the major and minor axis of the curves. The response at the central point depicts the highest degree of achievable catalase synthesis for that set of variables. Response surface curves were obtained by keeping the catalase production on the Z-axis against any two independent factors while holding the other independent factors at their ‘0’ level.

The 3D curve and a contour plot of calculated response surface obtained from the interaction between sucrose and yeast extract is presented in Fig. 5a. The highest catalase production of 39,400 IU/mg of cells was achieved when the concentration of yeast extract and sucrose was kept at 1.0 % (w/v) and 0.55 % (w/v), respectively. Thereafter, a decrease in enzyme activity was observed with the increase in either the yeast extract or sucrose. This may be due to the inhibitory effect of both nitrogen and carbon source at higher concentrations. The interaction between inoculum size and age of inoculum indicated that inoculum level above ‘0’ level (5 %, v/v) showed the best response with 6 h old inoculum culture for the catalase production (Fig. 5b). Further, response curves were also varied at different levels of BaCl_2_ along the axis which describes that there is a significant interaction between inoculum size and BaCl_2_. The response shown in Fig. 5c depicts the combined effect of above said variables in the fermentation media for catalase production (43,000 IU/mg of cells). A sharp increase in the catalase production was also achieved after 12 h of incubation by using computational tools as compared to that of 18 h at the flask level (Fig. 5d).

### Validation of the model

Catalase production as envisaged by the final quadratic model along with the corresponding observed values suggests that there is a good agreement between the predicted and experimental values. The optimum values for each factor [sucrose (0.55 %, w/v), yeast extract (1.0 %, w/v), BaCl_2_ (0.08 %, w/v), inoculum age (6 h), inoculum size (5 %, v/v) and fermentation time (12 h)] obtained by differentiation of quadratic model for achieving maximum catalase production were used for additional experiments to validate the accuracy of the model. The predicted optimal catalase production corresponding to these values was found to be 49,780 IU/mg of cells. The maximum catalase production of 48,900 IU/mg of cells obtained proves an excellent agreement between the predicted and experimental values, which further validates this statistical model. A considerable increase (118.11 %) in catalase production was obtained at the shake flask level using statistical tools.

### Scale up studies

**Fig. 6:**
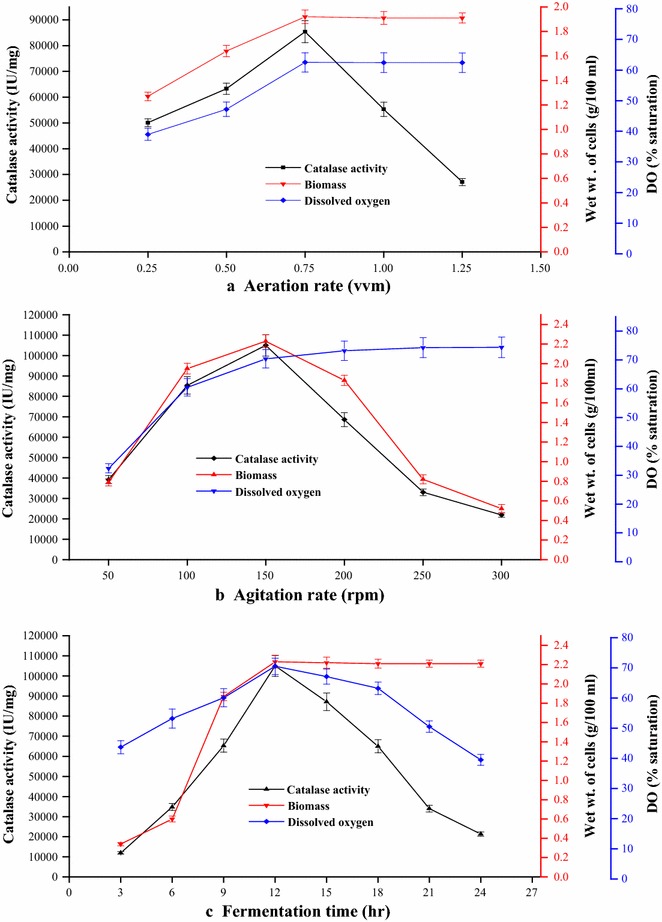
Influence of bioreactor parameters on catalase production from *Geobacillus* sp. BSS-7 **a** aeration rate, **b** agitation rate, **c** fermentation time

To further improve the catalase production on a pilot scale, the effect of some critical parameters such as agitation, air supply and fermentation time were investigated at bioreactor level. The key parameter in bioreactor studies i.e. the air supply was varied from 0.25 to 1.25 vvm to achieve the maximum enzyme yield. The optimum volume of air supply was found to be about 0.75 vvm with a dissolved oxygen concentration of 62.5 % saturation, at which maximum catalase production of 85,400 IU/mg of cells was obtained (Fig. 6a). Low biomass production and catalase synthesis at lower aeration rate (less than 0.75 vvm) was recorded due to oxygen limitation conditions. This major increase in catalase production might be due to continuous air supply to the bacterium because oxygen transfer rate is a critical factor that influences the overall cell metabolism. However, it was observed that high aeration rate above optimum level have caused metabolic shift in favour of cell growth which proved detrimental for enzyme synthesis. The low enzyme production at higher aeration beyond 0.75 vvm may also be due higher enzyme inactivation caused by irreversible oxidation of amino acid residues of the enzyme structure [30].

It was also observed that there was an increase in catalase production on increasing the rate of agitation up to 150 rpm (105,000 IU/mg of cells), and thereafter, enzyme production decreased significantly. An increase in dissolved oxygen concentration from 32.4 to 74.4 % saturation was also observed with enhanced agitation rate from 50 to 300 rpm (Fig. 6b). The higher growth and highest catalase synthesis was achieved at 150 rpm with a dissolved oxygen concentration of 70.4 % saturation. This might be due to continuous supply of air and even nutrient distribution facilitated by agitation. Two factors, namely oxygen supply and shear stress generally act in opposite direction, where oxygen supply enhances biomass production but shear stress lowers the enzyme productivity. The shearing effect preponderates the effect of oxygen supply at a higher agitation speed (>150 rpm) and induces cell disruption and enzyme inactivation. Hence, low biomass and low enzyme activity was observed at higher agitation rates beyond optimum level. Lower enzyme activity at a higher shear stress may also be due to its detrimental effect on enzyme structure [31].

Further, the effect of fermentation time was studied with an interval of 3 h up to 24 h of fermentation. It was found that the maximum catalase production (105,000 IU/mg of cells) was achieved after 12 h of incubation in the bioreactor (Fig. 6c). The decrease in enzyme production by bacterial cells on reaching the stationary phase after 12 h can be ascribed to nutrient depletion. A significant enhancement in catalase production (105,000 IU/mg of cells) was achieved in the bioreactor when compared to shake flask level (48,900 IU/mg of cells). This extraordinary enzyme production in a very short fermentation time of 12 h is a significant achievement, which has not been achieved by any method reported in the literature till date. A comparative analysis of methodologies involving catalase production from microorganisms (Table 4) clearly establishes that the developed methodology provides a simple and economical medium that employs only three constituents. Contrary to it, all the reported methodologies use complex multi component media for different microorganisms for synthesis of catalase [9, 13, 32].

**Table 4:** Comparison of intracellular catalase production from *Geobacillus* sp. BSS-7 with other microorganisms

Microbial source	Production media (g/l)*	Process parameters					Catalase production	Reference
		pH	Temp. (°C)	Time (h)	Inoculum size	Agitation (rpm)/stationary		
*Geobacillus* sp. BSS-7	Suc (5.5), YE (10.0), BaCl_2_ (0.8)	7.0	60	12	5 %, (v/v)	150	105000 ± 2563 IU/mg	Present study
*Rhizobium radiobacter* Strain 2-1	Pep (10), YE (5), NaCl (10), H_2_O_2_ (0.5)	(–)	30	20	(–)	130	30420 ± 1083 IU/mg	[8]
*Serratia* *marcescens* SYBC08	CSTL (33.8), citric acid (30)	7.0	30	40	4 % (v/v)	400	20289 IU/ml	[28]
*Exiguobacterium* *oxidotolerans* T-2-2^T^	Pep (8.0), YE (3.0), Sodium succinate (5.0), Aminolevulinic acid (2.0 mM), Tween 60 (1.0)	7.5	27	24	(–)	60	16000 IU/ml	[9]
*Psychrobacter piscatorii*	Pep (8), YE (3), NaCl (5), H_2_O_2_ (10 mM)	(–)	27	(–)	(–)	(–)	12000 IU/mg	[33]
*Bacillus halodurans* LBK 261	Glu (3), YE (5), Pep (5), KH_2_PO_4_ (1), NaCl (1.5), NaNO_3_ (10), CaCl_2·_2H_2_O (1.7), FeSO_4·_7H_2_O (1.3), MnCl_2·_4H_2_O (15.1), ZnSO_4·_7H_2_O (0.25), H_3_BO_3_ (2.5),CuSO_4·_5H_2_O (0.12), Na_2_MoO_4·_2H_2_O (0.12), Co(NO_3_)_2·_6H_*2*_O (0.23), H_2_SO_4_ (2.5 ml)	10	55	(–)	(–)	200	275 IU/g	[13]

*Glu* glucose; *Pep* peptone; *YE* yeast extract; *CSTL* corn steep liquor powder; *Suc* sucrose; (–) Data not available

## Conclusions

One factor at a time traditional approach and response surface methodology was applied for tailoring nutritional and process requirements of *Geobacillus* sp. BSS-7. A hyperproduction of catalase was achieved by customizing media components (sucrose 0.55 %, w/v; yeast extract 1.0 %, w/v; BaCl_2_ 0.08 %, w/v) and process variables (inoculum age 6 h; inoculum size 5 %, v/v and fermentation time of 12 h) at an optimum level. High similarity recorded between the predicted and experimental responses reflected the accuracy and applicability of statistical model used. A record 214.72 % increase in catalase production on implementing the bioprocess from the flask to bioreactor level proves the significance of multiple process variables in the studied fermentation process. The hyperproduction of catalase in a bioreactor is attributed to improved aeration and uniform distribution of nutrients. This is the first report on the hyper catalase production from any microbial source in a short incubation time using simple and economical media. The findings of this work would be useful for catalase production from *Geobacillus* sp. BSS-7 on a commercial scale to meet industrial demands.

## Methods

### Microorganism and culture conditions

*Geobacillus* sp. MTCC 5873, an extremophilic isolate of our laboratory (Enzyme Biotechnology Laboratory, Punjabi University, Patiala, INDIA), was grown in a medium (prepared in 50 mL distilled water) containing peptides digest of animal tissue (5.0 g/L), NaCl (5.0 g/L), beef extract (1.5 g/L) and yeast extract (1.5 g/L) for preparation of seed culture at 50 °C for 12 h on a rotary shaker inoculated with 5 % (v/v) inoculum culture. Further, a short term preservation of the isolate was carried on sterilized nutrient agar slants containing the aforementioned media supplemented with agar powder (15.0 g/L) by sub-culturing at fortnight intervals. The long term storage of the isolate was done at −80 °C in a 15 % glycerol solution.

### Shake flask optimization of medium constituents and process parameters

The production of intracellular catalase was carried out in a fermentation medium (50 mL) containing the yeast extract (0.5 %, w/v) and glucose (1.0 %, w/v) having pH of 7.0 on a rotary shaker (Certomat M, Germany) at 100 rpm for 24 h at 50 °C, unless otherwise specified. The individual effect of various carbon sources (glucose, fructose, sucrose, maltose, lactose, starch, mannose, raffinose, galactose, inoisotol) at 0.5 % (w/v) concentration was investigated to study their effect on catalase synthesis and biomass production.

Various nitrogen sources (Urea, NH_4_SO_4_, NH_4_NO_3_, NaNO_3_, KNO_3_, meat extract, yeast extract, malt extract, beef extract, tryptone and peptone) at 0.5 % (w/v) concentration were also added in the medium to study their influence on enzyme and biomass production. The effect of concentration of selected carbon and nitrogen source was also investigated. The medium was also supplemented with different metal salts (CaCl_2_, ZnSO_4_, CuSO_4_, MnSO_4_, BaCl_2_, MgSO_4_, FeSO_4_, NiSO_4_, CoCl_2_, HgSO_4_, AgNO_3_, KI, Na_2_SO_3_, and Na_2_MoO_4_) independently to study their influence on enzyme production. The effect of various bioprocess parameters such as pH of the medium, temperature, inoculum age and size, agitation rate, and fermentation time on catalase production was also investigated.

### Statistical design for selection of critical variables

A central composite rotatable design (CCRD) with six critical variables having 42 runs was used to determine the optimum combination of variables by studying their response patterns [34]. The selection of critical components (Table 5) of the medium was made on the basis of observations recorded using one variable at a time classical approach as discussed in previous section. The statistical analysis of the response patterns was made using the Design Expert 7.0.3 statistical software (Stat-Ease Inc., MN, USA). The experimental design with values in actual and coded form using Design Expert Program is listed in Table 5. The results of the final design were validated using analysis of variance (ANOVA) alongwith with Fischer test by testing its significant effect (P < 0.05).

**Table 5:** Levels of variables chosen for the experimental design

Symbols	Factors	Actual levels of coded factors		
		−1.000	0.00	1.000
A	Sucrose (%, w/v)	0.25	1.00	2.00
B	Yeast extract (%, w/v)	0.25	0.50	1.25
C	Barium chloride (%, w/v)	0.01	0.05	0.10
D	Inoculum age (h)	6.00	12.00	18.00
E	Inoculum size (%, v/v)	1.00	2.50	10.00
F	Time (h)	6.00	18.00	24.00

### Bioreactor studies

Bioreactor studies were carried out on a laboratory scale stirred tank reactor of 1.5 L capacity with working volume of 1.0 L (Biolab, B. Braun, Germany) having Ruston type impeller with six blades. Different parameters such as aeration (0.25–1.25 vvm), agitation (50–300 rpm) and fermentation time (3–24 h) were varied to achieve maximum production of catalase. To control foam formation, sterilized olive oil (0.002 %, v/v) was added at the start of process system.

### Recovery of catalase and enzyme assay

The biomass from the fermented broth was harvested by centrifugation (REMI, CPR-30 Plus) at 5000 g for 10 min at 4 °C for the extraction of intracellular catalase. The bacterial cells were disrupted (25 kHz, 10 min, pulse ON 25 s, OFF 05 s) by sonication (Vibra-Cell VCX 130, Newtown, USA) at 4 °C. The slurry was then centrifuged to separate the cell debris and supernatant thus obtained was analyzed for catalase activity. The estimation of catalase was carried out by the established method [35]. Catalase activity was calculated in terms of percent degradation of H_2_O_2_ and one unit (IU) of enzyme activity was expressed as an amount of enzyme that decomposes one micromole of H_2_O_2_ per minute under standard conditions.

## References

[1] BCC Research Report. Global markets for enzymes in industrial applications-Report code: BIO030H. http://www.bccresearch.com (2014). Accessed 14 Apr 2015.

[2] Shin DH, Choi YS, Cho YH (2008). Unusual properties of catalase A (KatA) of *Pseudomonas aeruginosa* PA14 are associated with its biofilm peroxide resistance. J Bacteriol.

[3] Chelikani P, Fita I, Loewen PC (2004). Diversity of structures and properties among catalases. Cell Mol Life Sci.

[4] Sooch BS, Kauldhar BS, Puri M (2014). Recent insights into microbial catalases: isolation, production and purification. Biotechnol Adv.

[5] Xenopoulos MA, Bird DF (1997). Effect of acute exposure to hydrogen peroxide on the production of phytoplankton and bacterioplankton in a mesohumic lake. Photochem Photobio..

[6] Fiedurek J, Gromada A (1997). Screening and mutagenesis of molds for improvement of simultaneous production of catalase and glucose oxidase. Enzym Microb Technol..

[7] Ko HS, Fujiwara H, Yokoyama Y, Ohno N (2005). Inducible production of alcohol oxidase and catalase in a pectin medium by *Thermoascus aurantiacus* IFO 31693. J Biosci Bioeng.

[8] Nakayama M, Nakajima-Kambe T, Katayama H, Higuchi K, Kawasaki Y, Fuji R (2008). High catalase production by *Rhizobium**radiobacter* strain 2-1. J Biosci Bioeng.

[9] Hanaoka Y, Yumoto I (2015). Manipulation of culture conditions for extensive extracellular catalase production by *Exiguobacterium**oxidotolerans* T-2-2^T^. Ann Microbiol.

[10] Wang H, Tokushige Y, Shinoyama H, Fujii T, Urakami T (1998). Purification and characterization of a thermostable catalase from culture broth of *Thermoascus**aurantiacus*. J Ferment Bioeng..

[11] Yumoto I, Yamazaki K, Kawasaki K, Ichise N, Morita N, Hoshino T, Okuyama H (1998). Isolation of *Vibrio* sp. S-1 exhibiting extraordinarily high catalase activity. J Ferment Bioeng.

[12] Puri M, Banerjee A, Banerjee UC (2005). Optimization of process parameters for the production of naringinase by *Aspergillus niger* MTCC 1344. Proc Biochem..

[13] Oluoch KR, Welander U, Andersson MM, Mulaa FJ, Mattiasson B, Hatti-Kaul R (2006). Hydrogen peroxide degradation by immobilized cells of alkaliphilic *Bacillus**halodurans*. Biocatal Biotransform.

[14] Gomaa OL, Momtaz OA (2006). Characterization of the hydrogen peroxide tolerating *Bacillus**maroccanus* type strain isolated from textile wastewater. Arab J Biotechnol..

[15] Takebe F, Hara I, Matsuyama H, Yumoto I (2007). Effects of H_2_O_2_ under low and high aeration level conditions on growth and catalase activity in *Exiguobacterium oxidotolerans* T-2-2T. J Biosci Bioeng.

[16] Khanna S, Srivastava AK (2005). Statistical media optimization studies for growth and PHB production by *Ralstonia eutropha*. Proc Biochem..

[17] Caridis KA, Christakopoulos P, Macris BJ (1991). Simultaneous production of glucose-oxidase and catalase by *Alternaria alternata*. Appl Microbiol Biotechnol.

[18] Levy E, Eyal Z, Hochman A (1992). Purification and characterization of catalase-peroxidase from the fungus *Septoria tritici*. Arch Biochem Biophys.

[19] Unlu AE, Takac S (2012). Investigation of the simultaneous production of superoxide dismutase and catalase enzymes from *Rhodotorula glutinis* under different culture conditions. Artif Cells Blood Substit Immobil Biotechnol.

[20] Sooch BS, Kauldhar BS. Influence of multiple bioprocess parameters on production of lipase from *Pseudomonas* sp. BWS-5. Braz Arch Biol Technol. 2013;56(5):711-21.

[21] Zeng HW, Cai YJ, Liao XR, Qian SL, Zhang F, Zhang DB (2010). Optimization of catalase production and purification and characterization of a novel cold-adapted Cat-2 from mesophilic bacterium *Serratia marcescens* SYBC-01. Ann Microbiol.

[22] Buckova M, Godocikova J, Zamocky M, Polek B (2010). Screening of bacterial isolates from polluted soils exhibiting catalase and peroxidase activity and diversity of their responses to oxidative stress. Curr Microbiol.

[23] Gudelj M, Fruhwirth GO, Paar A, Lottspeich F, Robra KH, Cavaco-Paulo A (2001). A catalase–peroxidase from a newly isolated thermoalkaliphilic *Bacillus* sp. with potential for the treatment of textile bleaching effluents. Extremophiles.

[24] Marchant R, Franzetti A, Pavlostathis SG, Tas DO, Erdbrugger I, Unyayar A, Mazmanci MA, Banat IM (2008). Thermophilic bacteria in cool temperate soils: are they metabolically active or continually added by global atmospheric transport?. Appl Microbiol Biotechnol.

[25] Marchant R, Banat IM, Rahman TJ, Berzano M (2002). What are high temperature bacteria doing in cold environments?. Trends Microbiol.

[26] Calandrelli V, Gambacotra A, Romano I, Carratore V, Lama L (2008). A novel thermo-alkali stable catalase–peroxidase from *Oceanobacillus oncorhynchi* subsp. *incaldaniensis*: purification and characterisation. World J Microbiol Biotechnol.

[27] Loewen PC, Switala J (1987). Purification and characterization of spore specific catalase-2 in *Bacillus**subtilis*. Biochem Cell Biol.

[28] Zeng HW, Cai YJ, Liao XR, Zhang F, Li YL, Zeng XK (2011). *Serratia marcescens* SYBC08 catalase isolated from sludge containing hydrogen peroxide shows increased catalase production by regulation of carbon metabolism. Eng Life Sci.

[29] Thompson VS, Schaller KD, Apel WA (2003). Purification and characterization of a novel thermo-alkali stable catalase from *Thermus**brockianus*. Biotechnol Prog.

[30] Cabiscol E, Tamarit J, Ros J (2000). Oxidative stress in bacteria and protein damage by reactive oxygen species. Int Microbiol..

[31] Calderbank PH, Moo-Young MB (1959). The prediction of power consumption in the agitation of non-newtonian fluids. Trans Inst Chem Eng.

[32] Wang W, Sun M, Liu W, Zhang B (2008). Purification and characterization of a psychrophilic catalase from antarctic *Bacillus*. Can J Microbiol.

[33] Yumoto I, Hirota K, Kimoto H, Nodasaka Y, Matsuyama H, Yoshimune K. *Psychrobacter piscatorii* sp. nov., a psychrotolerant bacterium exhibiting high catalase activity isolated from an oxidative environment. Int J Sys Evol Microbiol. 2010;60:205-08. 10.1099/ijs.0.010959-0 19648327

[34] Cochran WG, Cox GM (1992). In experimental designs.

[35] Sinha AK (1972). Colorimetric assay of catalase. Anal Biochem.

